# Mutations Located outside the Integrase Gene Can Confer Resistance to HIV-1 Integrase Strand Transfer Inhibitors

**DOI:** 10.1128/mBio.00922-17

**Published:** 2017-09-26

**Authors:** Isabelle Malet, Frédéric Subra, Charlotte Charpentier, Gilles Collin, Diane Descamps, Vincent Calvez, Anne-Geneviève Marcelin, Olivier Delelis

**Affiliations:** a Sorbonne Universités, UPMC Université Paris 06, INSERM, Institut Pierre Louis d’Epidémiologie et de Santé Publique (IPLESP UMRS 1136), Paris, France; b Department of Virology, AP-HP, Hôpital Pitié-Salpêtrière, Paris, France; c LBPA, ENS Cachan, CNRS UMR 8113, IDA, FR3242, Université Paris-Saclay, Cachan, France; d INSERM, IAME, UMR1137, Paris, France; e Université Paris Diderot, IAME, UMR1137, Sorbonne Paris Cité, Paris, France; f AP-HP, Hôpital Bichat, Laboratoire de Virologie, Paris, France; Medical School, University of Athens

**Keywords:** 3' PPT, dolutegravir, human immunodeficiency virus, integration

## Abstract

Resistance to the integrase strand transfer inhibitors raltegravir and elvitegravir is often due to well-identified mutations in the integrase gene. However, the situation is less clear for patients who fail dolutegravir treatment. Furthermore, most *in vitro* experiments to select resistance to dolutegravir have resulted in few mutations of the integrase gene. We performed an *in vitro* dolutegravir resistance selection experiment by using a breakthrough method. First, MT4 cells were infected with human immunodeficiency virus type 1 (HIV-1) Lai. After integration into the host cell genome, cells were washed to remove unbound virus and 500 nM dolutegravir was added to the cell medium. This high concentration of the drug was maintained throughout selection. At day 80, we detected a virus highly resistant to dolutegravir, raltegravir, and elvitegravir that remained susceptible to zidovudine. Sequencing of the virus showed no mutations in the integrase gene but highlighted the emergence of five mutations, all located in the *nef* region, of which four were clustered in the 3′ polypurine tract (PPT). Mutations selected *in vitro* by dolutegravir, located outside the integrase gene, can confer a high level of resistance to all integrase inhibitors. Thus, HIV-1 can use an alternative mechanism to develop resistance to integrase inhibitors by selecting mutations in the 3′ PPT region. Further studies are required to determine to what extent these mutations may explain virological failure during integrase inhibitor therapy.

## IMPORTANCE

Integrase strand transfer inhibitors (INSTIs) are increasingly used both as first-line drugs and in rescue therapy because of their low toxicity and high efficacy in both treatment-naive and treatment-experienced patients. Until now, resistance mutations selected by INSTI exposure have either been described in patients or selected *in vitro* and involve the integrase gene. Most mutations selected by raltegravir, elvitegravir, or dolutegravir exposure are located inside the catalytic site of the integrase gene, but mutations outside the catalytic site of the integrase gene have also been selected with dolutegravir. Following *in vitro* selection with dolutegravir, we report, for the first time, a virus with selected mutations outside the HIV-1 integrase gene that confer resistance to all integrase inhibitors currently used to treat patients, such as raltegravir, elvitegravir, and dolutegravir. Our observation may explain why some viruses responsible for virological failure in patients treated with dolutegravir did not show mutations in the integrase gene.

## INTRODUCTION

After its entry into the host cell, the HIV-1 RNA genome is converted into double-stranded DNA. This step, carried out by the reverse transcriptase, requires a different primer for the synthesis of each DNA strand; the first is a host-derived tRNA primer used to copy the viral RNA into a minus strand DNA, resulting in an RNA-DNA duplex, of which the RNA is degraded by RNase H, and the second is a 3′ polypurine tract (PPT) used as a primer for plus strand DNA synthesis (1). The purine-rich 3′ PPT is an essential conserved sequence element found within the RNA genomes of all retroviruses and is relatively resistant to RNase H cleavage, unlike most viral RNA sequences. The 3′ PPT is located at the PPT-U3 junction and consists of 15 nucleotides (5′ AAAAGAAAAGGGGGG 3′) (2). Specific and accurate removal of the RNA primers is crucial because it will define the ends of the linear viral DNA used for the subsequent reaction (1, 3).

After synthesis of the full-length linear viral DNA, it is integrated into the host cell chromatin through the action of the viral integrase (IN) enzyme, which catalyzes two reactions (3, 4). Within the cytoplasm, IN cleaves the conserved GT dinucleotides from the 3′ ends of the double-stranded HIV-1 DNA, generating CA-3′ OH DNA ends. The resulting processed 3′ DNA is used as a substrate for the integration process in a nucleoprotein complex called the preintegration complex (PIC). The nucleophilic agent for this reaction consists of the 3′ OH of the processed 3′ DNA end, leading to the covalent insertion of the viral DNA into the genome of the infected cell.

IN is an important target for the treatment of HIV infection because of its central role in HIV-1 replication. In 2007, IN was the last viral enzyme to emerge as a target for inhibitors to block HIV-1 replication. Integrase strand transfer inhibitors (INSTIs) are small molecules that bind to the active site of IN in the PIC context, causing it to disengage from the 3′ end of the viral DNA (4). Crystal structures of wild-type and mutant prototype foamy virus intasomes, an IN tetramer assembled on a pair of viral DNA ends, have been used to show that dolutegravir (DTG) enters deeper into the pocket vacated by the displaced DNA base, thus making closer contacts with viral DNA than those made by raltegravir (RAL) and elvitegravir (EVG). This suggests that DTG can readjust its position and conformation in response to structural changes in the active sites of RAL-resistant INSTIs (5, 6).

RAL and EVG were the first INSTIs to be used for the treatment of patients (7, 8). Failures with these inhibitors were observed and associated with the emergence of mutations selected in the IN gene, mostly involving three residues, N155, Q148, and Y143 (9–11). However, the virus in nearly half of the patients failing RAL or EVG treatment did not have mutations in the IN gene (12, 13). DTG belongs to the next generation of INSTIs, showing a greater genetic barrier to resistance and efficacy against viruses resistant to RAL and EVG (14). Thus, selection of resistance mutations under DTG is rare but exists and the mutations selected are similar to those described under RAL as Q148R/H, N155H, and G118R. But to date, no specific mutations leading to DTG resistance have been described (15). *In vitro* strategies using conventional resistance selection, including a gradual DTG concentration increase, showed that DTG has a resistance profile markedly distinct from those of RAL and EVG. Several mutations of nonpolymorphic residues such as S153, R263, and G118 have been described, but all were reported to confer low-level *in vitro* DTG phenotype changes (16–18).

As the classical way to select resistant viruses *in vitro*, using a gradually increase in the inhibitor concentration, have not allowed us to select high DTG resistance mutations so far, we decided to use a breakthrough selection experiment such as that already used for HIV-2 to select for EVG resistance (19). Our method consisted of cultivating HIV-1 Lai in the presence of a high and constant concentration of DTG, whereas the virus was already integrated into cells, allowing the constitutive production of viruses. This method allowed us to obtain a virus highly resistant to INSTIs with mutations selected outside the IN gene.

## RESULTS

### Methodology.

A DTG resistance selection experiment was performed by using a breakthrough method. Initially, MT4 cells were infected with the wild-type Lai virus (50 ng of p24 antigen/10^6^ cells) without DTG to allow integration of the virus. Twenty-four hours later, cells were washed twice with phosphate-buffered saline to remove unbound virus and suspended in fresh medium in the presence of 500 nM DTG, approximately 170 times the 50% effective concentration (EC_50_) of DTG. At the same time, the virus was also cultured without DTG. This method, using a strand transfer inhibitor at a high concentration, has already been used for HIV-2 to select resistance to EVG (19). DTG was added to the medium every 3 days to maintain a constant high concentration. Cells were also regularly removed, and the medium was partially replaced. After approximately 60 days of culture, cell death occurred and it became necessary to add uninfected cells to maintain the culture. The culture was maintained beyond the initial viral breakthrough until 118 days. The aim of this study was to characterize the virus capable of replicating in the presence of this high DTG concentration. We named it the 9053 virus.

### Phenotypic analysis of the 9053 virus (culture at day 80).

We tested RNAs extracted from supernatants for the presence of mutations in the IN gene throughout the DTG breakthrough experiment. Genotypic analysis showed that no mutations were selected in the IN gene through the end of the selection period of 118 days, whereas an obvious viral breakthrough occurred on day 60. We tested the supernatant containing the 9053 virus at day 80 for phenotypic resistance to DTG, EVG, RAL, and zidovudine (AZT), comparing it to that of the wild-type Lai virus.

**FIG 1:**
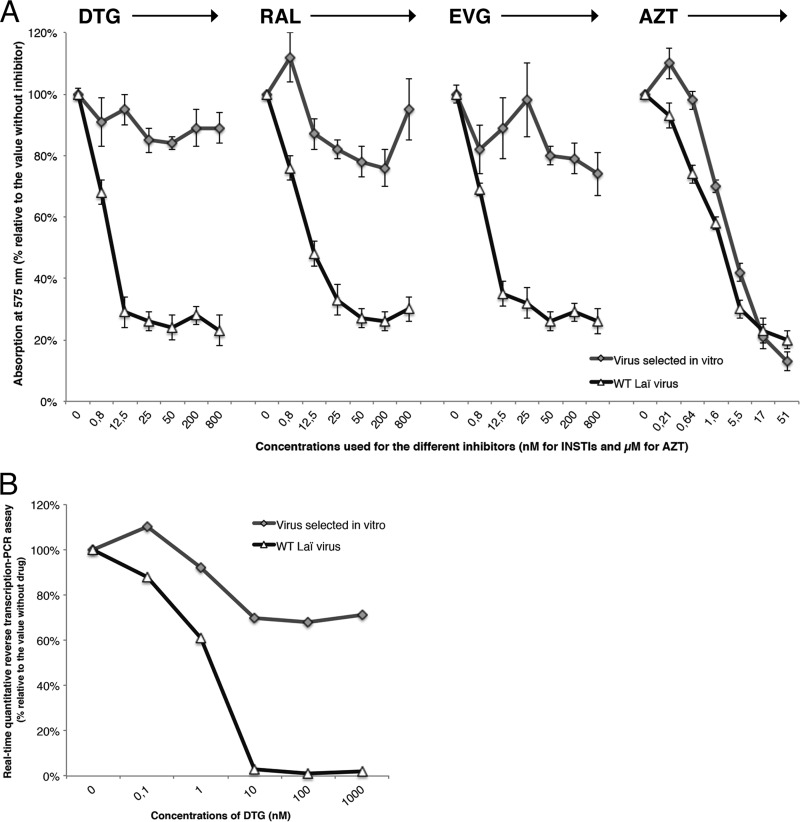
Phenotypic analysis of the 9053 virus selected under DTG compared to the wild-type Lai virus. Cells were infected in 96-well plates with wild-type (WT) or mutant virus. (A) HeLa P4 cells infected in triplicate with increasing concentrations of the inhibitor DTG, RAL, EVG (0 to 800 nM), or AZT (0 to 51 μM). (B) PBMCs infected in quadruplicate with increasing concentrations of DTG (0 to 1,000 nM).

**FIG 2:**
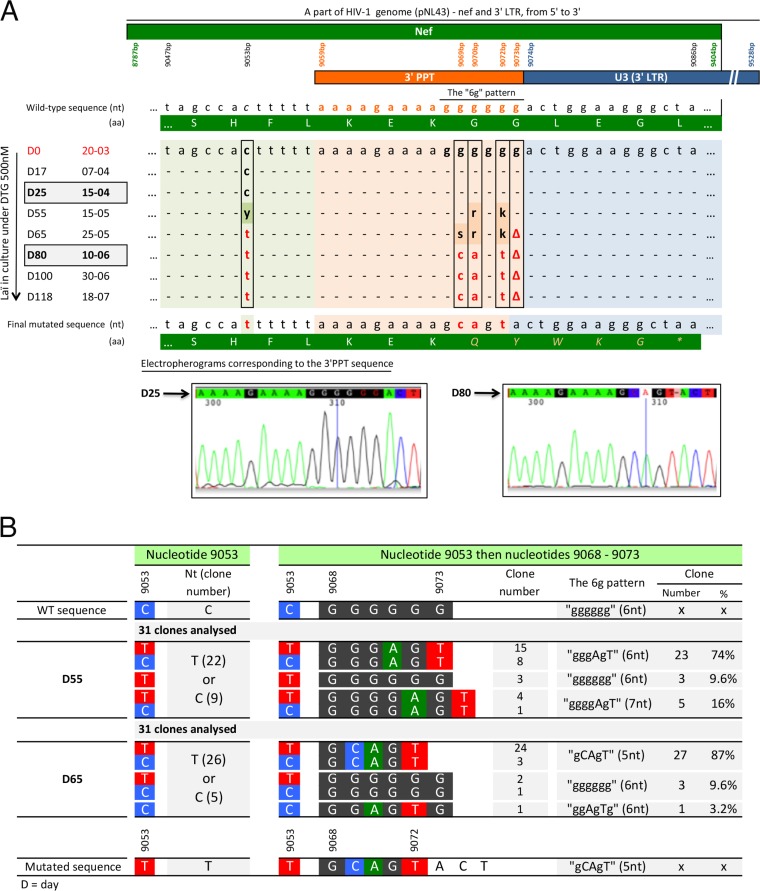
Sequencing of the 3′ PPT region of viruses during DTG selection. (A) Analysis of samples throughout all selection steps. nt, nucleotides; aa, amino acids. (B) Clonal analysis of supernatants on days 55 and 65.

Phenotypic analysis of the wild-type virus with HeLa P4 cells allowed us to calculate the EC_50_ of each inhibitor (3 nM for DTG, 10 nM for RAL, and 5 nM for EVG). The 9053 virus lost approximately 50% of its infectivity relative to the wild-type Lai virus but showed a high level of resistance to all three INSTIs tested, with fold changes (FCs) of >270 for DTG, >80 for RAL, and >160 for EVG (Fig. 1). In addition, both viruses (9053 and the wild type) were fully susceptible to AZT (EC_50_, 2 μM) (Fig. 1). Finally, we also tested for the DTG resistance phenotype by using a peripheral blood mononuclear cell (PBMC) assay that showed an FC of >300 (Fig. 2).

### Genotypic analysis of the 9053 virus (day 80).

The resistance of the 9053 virus to the INSTIs in the absence of mutations in the IN gene led us to explore the entire genome (5′ long terminal repeat [LTR], gag, protease, reverse transcriptase, vif, vpr, tat, rev, vpu, gp120, gp41, nef, and 3' LTR) of the 9053 and Lai viruses cultured in parallel without DTG and compared their sequences with those of the wild-type Lai virus. There were no differences between the two genomes except in the *nef* gene, where there were several modifications clustered in the 3′ PPT region of the 9053 virus. One mutation was located six nucleotides upstream of the 3′ PPT (at nucleotide 9053, numbered relative to the pNL43 sequence), and three mutations and one deletion were detected inside the GGGGGG 3′ PPT pattern (at nucleotides 9069, 9070, 9072, and 9073). The resulting sequence was A_9053_C and 5′AAAAGAAAAG(G_9069_C)(G_9070_A)G(G_9072_T)(G_9073_deleted). Deletion of one nucleotide in the 3′ PPT involved a change in the open reading frame of the *nef* gene that generates a stop codon located 4 amino acids downstream of the deletion. Consequently, the nef protein would be shortened from 206 to 99 amino acids.

### Sequence analysis of the 3′ PPT (eight samples from day 0 to day 118).

We retrospectively sequenced the 3′ PPT region of samples taken throughout the passages under DTG selection (Fig. 2A). The first mutations were detected after approximately 2 months of culture, at day 55. More precisely, the analysis showed a mixture of sequences at days 55 and 65 (Fig. 2A) at the mutated positions described above, reflecting a mixture of viruses in the culture. By day 80 (Fig. 2A), we detected only one virus, which carried the mutated GCAGT pattern. There were no further changes in the sequence from day 80 to the end of the experiment at day 118, showing that the mutations were highly stable.

### Clonal analysis of the 3′ PPT (two samples, days 55 and 65).

We cloned amplified DNA obtained at days 55 and 65 (Fig. 2B). The clone from day 55 had a mixture of three patterns, GGGAGT (74%), GGGGAGT (composed of seven nucleotides; 16%), and wild-type GGGGGG (9.6%). At day 65, we detected a mixture of two patterns, GCAGT (87%) and wild-type GGGGGG (9.6%), whereas GGGAGT, which was present at day 55, had completely disappeared.

### Infection of MT4 cells by the 9053 virus (day 80).

**FIG 3:**
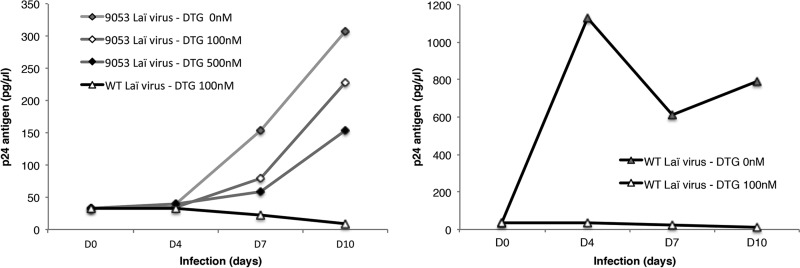
The 9053 virus is able to replicate in the presence of DTG. Viruses were used at a level of 50 ng of p24 antigen/10^6^ MT4 cells in the presence of DTG at different concentrations (0, 100, and 500 nM). The p24 antigen levels under the different conditions of the experiment were monitored. WT, wild type.

We tested the ability of the 9053 virus to infect new cells and replicate in the presence of DTG (Fig. 3). The wild-type virus did not replicate in the presence of 100 nM DTG, whereas the 9053 virus showed a gradual increase in p24 antigen production from the beginning until day 10. The increase from baseline p24 production depended on the concentration of DTG included in the medium (9.3-fold at 0 nM, 6.9-fold at 100 nM, and 4.6-fold at 500 nM), whereas in the absence of the inhibitor, the wild-type virus attained maximum replication at day 4 with a large increase (34-fold) in p24 antigen production (Fig. 3).

### Analysis of the virus derived from mutagenesis.

**FIG 4:**
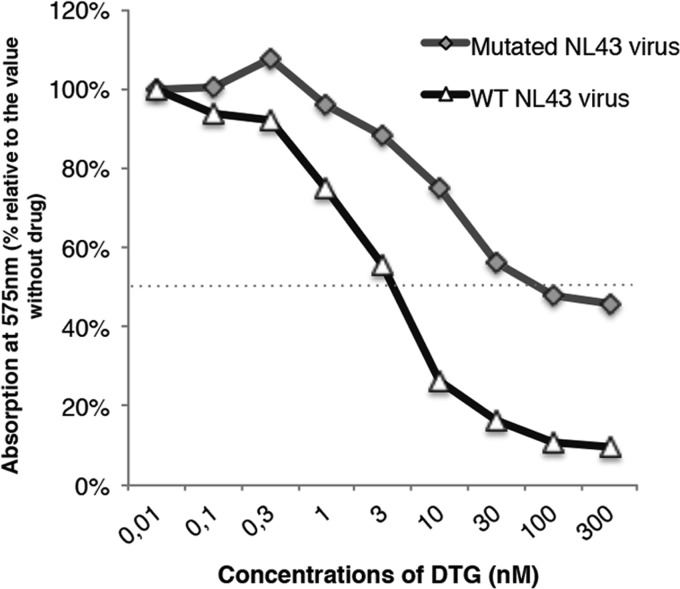
The NL43 virus carrying all of the mutations described in the 9053 virus was resistant to DTG. Both the wild-type and 9053 viruses were used to infect HeLa P4 cells in triplicate in 96-well plates in the presence of increasing concentrations of DTG (0 to 300 nM). WT, wild type.

**FIG 5:**
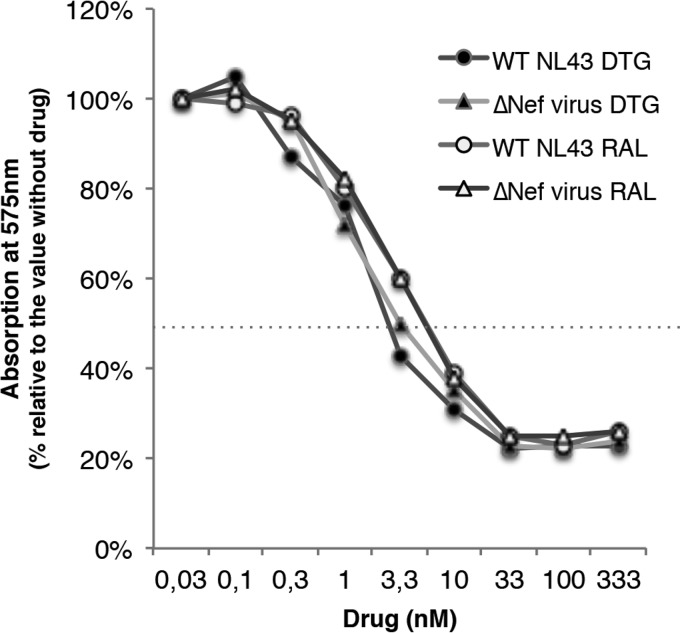
Susceptibility of wild-type and ΔNef NL43 viruses to DTG and RAL. All data shown are mean values ± standard errors (error bars) of three independent experiments. WT, wild type.

All of the mutations described in the 9053 virus were introduced into the pNL43 backbone by site-directed mutagenesis, and the corresponding virus was produced. The introduction of these mutations resulted in a loss of approximately 90% infectivity relative to the wild-type virus. Nevertheless, the mutant was far more resistant to DTG (EC_50_ of 80 nM) than the wild-type virus was (EC_50_ of 3.5 nM), with an FC of 23 (Fig. 4). The analysis of the NL43 ΔNef virus showed a comparable loss of infectivity (around 83%), and phenotypic analysis showed that, like the wild-type virus, it was sensitive to RAL and DTG (EC_50_s, 2.7 and 1.4 nM, respectively) (Fig. 5).

## DISCUSSION

To date, all studies that have focused on resistance to INSTIs, both *in vitro* and *in vivo*, have shown that mutations conferring resistance to INSTIs are selected within the catalytic site of IN (10, 20–22), which prevents binding of the inhibitor and restores the capacity of IN to fulfill its role of integrating the viral DNA into the chromosomal DNA. However, the virus of nearly half of the patients who fail RAL or EVG therapy do not have mutations in the IN gene (12, 13) and this proportion is even higher for DTG, suggesting the possibility of an alternative resistance mechanism.

We focused on DTG, an INSTI for which the mechanism of resistance is still poorly understood and which shows a higher genetic barrier than RAL and EVG in patients (20). The classical *in vitro* method to select viruses that are resistant to antiviral compounds such as strand transfer inhibitors is to gradually increase the concentration of the inhibitor in the cell medium. In the case of DTG, this method has led to a few mutations that confer low-level resistance, such as the R263K mutation (23). The *in vitro* approach used a breakthrough method consisting of maintaining a constant high DTG concentration, whereas the Lai virus was already integrated into the chromosome of MT4 cells. This method allowed the constitutive production of viruses able to enter new cells but unable to integrate because of the presence of DTG. Recovery of viral replication under these conditions required the production of a virus resistant to DTG in the culture supernatant. After approximately 60 days of culture, we succeeded in selecting a virus resistant to DTG, as well as RAL and EVG. Surprisingly, we found no mutations in the IN gene but rather five located in the *nef* region, one mutation six nucleotides upstream of the 3′ PPT and four other changes clustered in the 3′ end of the 3′ PPT, inside the G tract, resulting in GCAGT instead of GGGGGG. The analysis of Nef sequences available in the Los Alamos HIV-1 database, representing a total of 23,874 sequences, showed that the motif was highly conserved, with each G considered individually showing variability on the order of 0.05%.

The location of these mutations in a highly conserved region is very surprising, and the mechanism involved in this change remains unknown. The disruption of the 3′ PPT could lead to modification of the reverse transcription (RT) process, resulting in linear DNA that is no longer fully compatible with integration, explaining the decrease in infectivity. This nonconventional linear DNA could impair DTG binding, explaining the resistance to DTG. In addition, the deletion of one nucleotide induced an interruption in the reading frame of the *nef* gene leading to a stop codon located 5 amino acids downstream from the 3′ PPT, resulting in a nef protein with its C-terminal portion truncated. Patients infected with *nef*-defective HIV-1 show strongly attenuated viral replication and pathogenesis (24–26). Although numerous activities of the nef protein have been identified *in vitro*, nef appears to be dispensable for viral replication in cell culture (27). Results showed that the truncated nef protein alone plays no role in the resistance of the virus to DTG and RAL. Nevertheless, the hypothesis that this truncated protein would play a role in resistance to DTG when associated with mutations selected in 3′ PPT should be investigated.

Although we do not have an explanation for the mechanism used by the virus to escape DTG, this virus is clearly resistant to all currently used INSTIs. Indeed, phenotypic analysis showed a high FC in resistance to INSTIs, especially DTG (>270), whereas the virus is still susceptible to AZT, thus behaving normally with respect to inhibitors of targets other than IN. In addition, introduction of all of the mutations detected in the 9053 virus into the pNL43 backbone was sufficient to establish DTG resistance (FC, 23). We have thus demonstrated, for the first time, that selection of mutations outside the IN gene could be involved in INSTI resistance. Already, resistance mutations located outside the catalytic site of the enzyme, at positions 262 and 263, are specific to DTG, and similarly, it has been shown that mutations involved in protease inhibitor resistance can be selected outside the target gene, in the gag substrate cleavage sites (28, 29).

Discrepancies between the FCs (23 for pNL43 versus 270 for the 9053 virus) can be explained by the use of two different backbones (Lai versus pNL43). It would be necessary to use a plasmid closer to the Lai sequence for which the corresponding virus would have similar infectivity to compare the impact of the mutations identified.

Achieving such high resistance to DTG is novel. Studies concerning mutations specifically selected *in vitro* under DTG, all located in the IN gene, showed relatively low resistance to DTG when introduced into the pNL43 backbone, such as S153Y (FC, 2.5), R263K (FC, 11.2), and G118R (FC, 10) (16–18). Even the G140S Q148H double mutant, selected under RAL, with an FC in RAL resistance of 130, has an FC in DTG resistance of only 2.5 (16). Nevertheless, a clinically derived virus producing IN carrying G118R, which was selected in patients, had an FC of >100 (21). We have thus previously shown that the context in which the mutation is tested can play an important role in the evaluation of its contribution to resistance (21). Overall, the virus selected in this work shows a new ability to resist DTG.

In addition, the 9053 virus is still infectious, as shown by its ability to infect new cells, even if replication was quantitatively slowed by the presence of DTG, at least in the first days of infection. The production of p24 antigen by the 9053 virus was not due to the subpopulation of wild-type virus used for the initial infection, still present in the supernatants of the resistant virus, because the wild-type virus was completely impaired by DTG. Our data clearly show that this virus was able to replicate at a high DTG concentration.

We highlight a new mechanism of HIV-1 replication in the presence of DTG. As no mutation in the IN gene was selected, DTG can still bind to the active site of IN in the same way as the wild-type virus, which is completely inhibited by 100 nM DTG. There are two possibilities that could explain the replication of the mutant; i.e., (i) the virus is not integrated into cellular DNA and replication involves only unintegrated viral DNA (20) or (ii) the viral genome is integrated by an independent mechanism. Interestingly, no integrated viral DNA was quantified during the time course of the experiment using the mutant under DTG. This could be explained by the fact that no mutation has been highlighted in the IN gene and the use of a high DTG concentration that efficiently inhibits HIV-1 integration. The amount of viral DNA integrated under this condition could be below the detection limit of the method used because of the weak replication of the virus compared to that of the wild type. This hypothesis is under investigation, and further experiments are needed to explain the mechanism of replication of the mutant. Irrespective of the mechanism, replication of the 9053 virus is much slower than that of the wild-type virus. The sequence found in our mutant highlights a specific sequence found at the LTR-LTR junction of two-LTR circles (30, 31). This activity was also described for foamy virus (32). However, cleavage at this position (the boundary between the 3′ PPT and the 3′ LTR) is improbable. Indeed, the specific cleavage of this sequence is found only under conditions without an INSTI. Furthermore, this cleavage would lead to virus integrated without the 3′ LTR, leading to a defect in viral particles.

The *in vitro* selection of this virus, which is resistant to DTG by an unprecedented mechanism, was based on a DTG concentration of 500 nM. This is significantly higher than the 50% inhibitory concentration of DTG (2.7 nM) (16) but on the same order as the DTG concentration in human plasma when the drug is administered once or twice daily (33). Understanding the mechanism used by this virus to escape DTG is an important challenge that will shed further light on the astonishing ability of HIV-1 to adapt to DTG therapy without having to select mutations in the IN gene.

DTG is now widely used in the treatment of patients infected with viruses either with or without INSTI resistance mutations. The *in vitro* identification of a novel mechanism of DTG resistance involving mutations outside the IN gene warrants further studies to determine the extent to which mutations within the 3′ PPT could explain virological failures of DTG therapy in the absence of selected mutations in the IN gene.

## MATERIALS AND METHODS

### Cells.

293T and HeLa P4 cells were cultured in Dulbecco’s modified Eagle’s medium supplemented with 10% heat-inactivated FBS (fetal bovine serum). MT4 cells were cultured in RPMI with 10% FBS. All cells were maintained at 37°C in 5% CO_2_.

### Virus derived from mutagenesis.

The HIV-1 pNL43 plasmid carrying all of the mutations detected in the 3′ PPT, as well as pNL43ΔNef (with the G deletion at bp 9073), was obtained by site-directed mutagenesis with the QuikChange II XL site-directed mutagenesis kit (Agilent) in accordance with the manufacturer’s instructions. Virus was prepared with Lipofectamine reagent (Thermo Fisher) by transfecting 293T cells. Forty-eight hours posttransfection, the viral supernatant was harvested and frozen at −80°C. The titer of the supernatant was determined by quantifying HIV-1 p24 antigen with the VIDAS instrument (BioMérieux).

### Phenotypic analysis.

Single-cycle titers of the virus were determined in HeLa P4 cells in which the expression of β-galactosidase was induced by the HIV Tat protein and quantified 48 h postinfection with chlorophenol red‐-β‐d‐galactopyranoside substrate by using the absorbance at 575 nm. Briefly, cells were infected in triplicate in 96-well plates with wild-type or mutant virus (equivalent of 7 ng of p24 antigen) containing increasing concentrations of the inhibitor DTG, RAL, EVG (0 to 800 nM), or AZT (0 to 51 µM). Phenotypic susceptibility to DTG was evaluated by the Agence Nationale de Recherche sur le Sida (ANRS) PBMC method as previously described (34). Briefly, cells were placed in 96-well plates containing six serial dilutions of DTG (0 to 1 000 nM). Each dilution was tested in quadruplicate. On day 3, the supernatant was collected and the 50% tissue culture-infective dose was assessed by measuring the number of HIV RNA copies in the supernatant with a real-time quantitative RT-PCR assay (35).

### Viral infection.

MT4 cells were newly infected. Cells were preincubated with RPMI alone (DTG at 0 nM) or containing DTG at a final concentration of 100 or 500 nM for 2 h and then infected with the 9053 Lai virus from supernatant day 80 or wild-type Lai virus, both at a level of 30 ng of p24 antigen (virus equivalent)/10^6^ cells. The drug was added every 3 days to maintain a high concentration of the inhibitor in the medium to prevent putative integration from two-LTR circles as already shown (30). An aliquot of supernatant was regularly taken, and the p24 antigen content was quantified.

### Amplification, sequencing, and cloning from culture supernatants.

The RT-PCR assay was performed with the Transcriptor One-Step RT-PCR kit (Roche Life Science). Several genes were studied with the different primer sets (forward primer, FP; reverse primer, RP) listed here and numbered relative to the HxB2 sequence: 5′ LTR, FP_(1−23)_/RP_(991−1021)_; gag, FP_(734−754)_/RP_(2294−2315)_; protease, FP_(2071−2100)_/RP_(2705−2735)_; reverse transcriptase, FP_(2480−2499)_/RP_(3514−3546)_ and FP_(3208−3236)_/RP_(4038−4069)_; RNase H, FP_(3696−3715)_/RP_(4380−4399)_; IN, FP_(4007−4026)_/RP_(5251−5270)_; vif, FP_(4899−4922)_/RP_(5765−5786)_; vpr, FP_(5431−5453)_/RP_(5970−5988)_; tat-rev-vpu, FP_(5611−5639)_/RP_(6423−6443)_ and FP_(7634−7653)_/RP_(8285−8304)_; gp120, FP_(5833−5857)_/RP_(6553−6580)_ and FP_(6423−6443)_/RP_(7831−7849)_; gp41, FP_(7634−7653)_/RP_(9010−9038)_; nef-3′ LTR, FP_(8688−8713)_/RP_(9607−9632)_. Purified RT-PCR products were directly sequenced for bulk genotyping by using a cycle sequencing reaction with the BigDye Terminator kit (Applied Biosystems). Furthermore, some of the purified RT-PCR products were cloned with the TOPO TA Cloning kit (Invitrogen) and 31 clones were reamplified by PCR for sequencing. All of the sequences obtained were analyzed with the CodonCode Aligner software.
